# Influence of Polyol/Crosslinker Blend Composition on Phase Separation and Thermo-Mechanical Properties of Polyurethane Thin Films

**DOI:** 10.3390/polym12030666

**Published:** 2020-03-17

**Authors:** Said Arévalo-Alquichire, Maria Morales-Gonzalez, Kelly Navas-Gómez, Luis E. Diaz, José A. Gómez-Tejedor, María-Antonia Serrano, Manuel F. Valero

**Affiliations:** 1 Energy, Materials and Environmental Group, GEMA, Faculty of engineering, Universidad de La Sabana, 140013 Chía, Cundinamarca, Colombia; saidaral@unisabana.edu.co (S.A.-A.); kelly.navas@unisabana.edu.co (K.N.-G.); 2 Doctoral program of biosciences, Universidad de La Sabana, 140013 Chía, Cundinamarca, Colombia; 3 Bioprospecting Research Group, GIBP, Faculty of engineering, Universidad de La Sabana, 140013 Chía, Cundinamarca, Colombia; luisdb@unisabana.edu.co; 4 Centre for Biomaterials and Tissue Engineering, CBIT, Universitat Politècnica de València, Camino de Vera s/n, 46022 Valencia, Spain; jogomez@fis.upv.es (J.A.G.-T.); mserranj@fis.upv.es (M.-A.S.); 5 Biomedical Research Networking Center in Bioengineering, Biomaterials and Nanomedicine (CIBER-BBN), 46022 Valencia, Spain

**Keywords:** polyurethane, polyol, crosslinker, phase separation, thermo-mechanical properties

## Abstract

Polyurethanes (PUs) from Polyethylene glycol (PEG) and polycaprolactone diol (PCL) and a crosslinker, Pentaerythritol (PE), were synthetized with isophorone diisocyanate (IPDI). In this study, we investigated the effect of polyol and crosslinker composition on phase separation and thermo-mechanical properties. The properties were studied through dynamic mechanical analysis, X-ray scattering, atomic force microscopy (AFM), and thermogravimetric analysis (TGA). The results showed changes in PUs properties, microphase structure, and separation due to the composition of polyol/crosslinker blend. So, the largest concentration of PE produced multimodal loss factor patterns, indicating segment segregation while PUs with a PEG/PCL = 1 displayed a monomodal loss factor pattern, indicating a homogeneously distributed microphase separation. Additionally, the increase of the PEG concentration enhanced the damping capacity. On the other hand, agglomeration and thread-like structures of hard segments (HS) were observed through AFM. Finally, the thermal behavior of PUs was affected by chemical composition. Lower concentration of PE reduced the crosslinking; hence, the temperature with the maximum degradation rate.

## 1. Introduction

Polyurethanes (PUs) are a special and outstanding group of polymers with a wide range of applications owing to their particular physical and chemical properties, such as automotive, construction, furniture, insulation, textile, [1] and biomedical devices. PUs can be easily synthetized by the reactions of polyol with isocyanate and chain extenders or crosslinkers, creating a segmented structure composed of soft polyol chains and hard segments (HS), urethane bonds, isocyanate, and a chain extender or crosslinker. Physio-chemical and mechanical properties can be tuned based on the nature and structure of monomers [2]. Furthermore, microphases or domains composed by HS and soft segments (SS) are driven by thermodynamic incompatibility resulting in insolubility between HS and SS [3]. HS and SS can crystallize and become driving forces for phase separation [4]. This phase separation can lead to property changes because SS are responsible for the softness, flexibility, and rubbery behavior, while HS are related to stiffness and mechanical behavior. The elastomeric behavior of PUs results from embedding HS in SS, where the HS acts as crosslinker due to hydrogen bonding [5]. Hence, the segregation of HS and SS mediate PU properties. Aforesaid segregation can be studied by several microscopic, spectroscopic and crystallographic techniques like Atomic force microscopy (AFM), infrared spectroscopy (IR), both small (SAXS) and wide (WAXS) angle X-ray scattering.

Previous research has described this segregation as a function of the composition and chemical structure of monomers, and its effect on PU properties. e.g., Klinedinst, D. et al. [4] studied the effects of varying the SS molecular weight and overall HS content thermoplastic segmented PUs. They used AFM and dynamic mechanical analysis (DMA) to identify a thread-like microphase separated structure in chain-extended systems. A higher polyol molecular weight increased the partial crystallization of SS at lower temperatures than room temperature, and larger SS/HS incompatibility, which induced greater microphase separation and a larger storage modulus plateau magnitude. The larger HS mass also increased the temperature at which HS melting occurred, broadening the storage modulus plateau.

Some works have focused on the study of the chain extender or crosslinker structure. Like, Kim, H-N., et al. [1] who compared PU microphases with three chain extenders: isosorbide (ISB), isomannide (IMN), and 1,4-butanediol (BD). According to the SAXS results, the scattering widths of IMN and ISB-based PUs were larger than those of BD-based PUs, indicating that the HS domain sizes in the IMN and ISB-based PUs were smaller. It also demonstrated strong HS bonding in the IMN-based PUs despite their small domain size. Additionally, ISB and IMN chain extenders conferred more thermal reversibility to PUs than did BD. The ISB-based PU showed superior mechanical properties compared with those of BD and IMN-based PUs. Around the crosslinker, Ordon K. et al. [6] studied some PUs synthetized with BD as the chain extender and starch as the crosslinker together with hydroxyapatite. A PU thermogravimetric analysis (TGA) indicated the enhancement of PU thermal stability with an increase in the starch and hydroxyapatite content caused by crosslinking the structure.

Likewise, other authors have been working on the study of phase mixing and crystallization using different polyols nature and length. e.g., reduction of polyol length or molecular weight in polycaprolactone diol based PUs reduce the crystallization of soft segment [7]. Also, mixing polyhydroxyalkanoates (PHA) diol and polyethylene glycol (PEG) produce a change in the crosslinking and the increase of PHA produces a crystalline region on Pus [8]. While, PEG incorporation in Poly(tetra-methylene glycol) (PTMG) PUs drive different phenomena according to the molecular weight, PEG of 200 g/mol did not differs from pure PTMG based PUs, while 2000 g/mol produces the best phase separation.

In the past, we studied the influence of polyol crosslinker composition on polyurethane properties through a mixture design coupled with a surface response analysis [2]. There, we studied the physic-chemical and mechanical properties of PUs based on polycaprolactone diol and polyethylene glycol, as polyols, and pentaerythritol, as crosslinkers. We obtained mathematic models describing PUs properties as a function of composition. In this work, we studied the phase separation and thermomechanical properties of the same PU compositions to enhance comprehension of PUs performance.

## 2. Materials and Methods

### 2.1. Materials

PEG (Av. Mn~1000 g/mol) was purchased from Merck KGaA (Darmstadt, Germany). PCL (Average molecular weight (Av. Mn) ~2000 g/mol), IPDI, and N,N-dimethylformamide (DMF) were purchased from Sigma-Aldrich (St. Louis, MO, USA). PE was obtained from Alfa Aesar (Heysham, UK).

### 2.2. Synthesis of Polyurethanes

In this study, PEG and PCL were used as polyols, PE was used as the crosslinker, and IPDI was used as the isocyanate. PUs were synthetized by two-step polymerization as reported in previous work [2]. Briefly, PCL and PEG were dissolved in 10 mL of DMF, and the calculated amount of IPDI was added to the mixture. After reacting for 15 min at 70 °C, a second solution, PE in 10 mL of DMF, was added. The reaction was maintained at 100 °C for at least 5 h to allow polymerization and solvent evaporation. The IPDI amount was calculated using an NCO/OH ratio equal to 1. The sum of the polyol and crosslinker mols were used as OH variables in the calculations.

Thin films were casted on a glass surface using an Elcometer 3580 Casting Knife Film Applicator (Elcometer Ltd., Manchester, UK) with a gap of 150 µm. Afterward, the glass was transferred to an oven with a temperature of 110 °C for 12 h to allow for PU curing. The films were then peeled by soaking the glass in water and then mechanically extracting the films with a palette knife.

Eight polyol/crosslinker blends were studied, and their composition was defined by a mixture design detailed in previous research [2]. The blend compositions are presented in Table 1. The blends were named based on their composition in the order PEG-PCL-PE. For example, PU 0-90-10 was synthetized using 0% w/w of PEG, 90% w/w of PCL, and 10% w/w of PE.

A theoretical HS content was calculated based on the equation described by Lee et al. [9], which relates the number of isocyanate and chain extenders to the number of total components. HS was assumed to be IPDI and PE. Calculations were made using the following equation:

*%HS* (wt %) = 100 × (*W_IPDI_* + *W_PE_*)_/_(*W_IPDI_* + *W_PE_* + *W_PCL_* + *W_PEG_*) (1)

where *W_IPDI_*, *W_PE_, W_PCL_* and *W_PEG_* are the masses of IPDI, PE, PCL, and PEG, respectively.

### 2.3. Chemical Structure

The chemical structure and synthesis were evaluated using Fourier transform infrared spectroscopy (FTIR) with a Cary 630 FTIR spectrometer with a diamond attenuated total reflection (ATR) accessory (Agilent, Santa Clara, CA, USA). The spectra were recorded in a range of 650 cm^−1^ to 4000 cm^−1^ with an average of eight scans and a resolution of 2 cm^−1^. At least three spectra were recorded per sample.

The degree of phase separation (DPS) and hydrogen bounding index (HI) was calculated based on equations described by Amrollahi et al. [10], which are shown below:

DPS = −NH_3430_/(−NH_3430_ + −NH_3330_) (2)

HI = −C = O_1703_/−C = O_1730_ (3)

where *−NH_3430,_ −NH_3330,_ −C = O_1730,_ −C = O_1703_* are the absorbance at 3430, 3330, 1730, 1703 cm^−^^1^, respectively. Which corresponds to the free −NH, hydrogen bounded -NH, free −C = O, and hydrogen bounded groups, respectively. Before calculation, the absorbances were normalized in terms of 1700 cm^−1^ absorbance.

### 2.4. Thermo-Mechanical Properties and Morphology

A dynamic mechanical analysis (DMA) was performed in a DMA 8000 (Perkin-Elmer, Waltham, MA, USA) adapted with a tensile clamp. The equipment was set up with a frequency of 1 Hz, deformation of 0.05 mm, temperature range from −100 °C to 180 °C, and heating rate of 3 °C/min. The storage modulus, loss modulus, and Tan δ were recorded. Rectangular samples 9 mm in length, 5 mm in width, and 0.5 mm in thickness were measured.

PUs surface was studied using a Multimode 8 atomic force microscope (BRUKER, Billerica, MA, USA). The tapping mode was employed in the air with a 250 µm silicon tip. Topography and phase images were obtained.

X-ray scattering measurements were taken in a Panalytical X-Per Pro (Malvern, Malvern Panalytical, UK). Both SAXS and wide-angle X-ray scattering (WAXS) were used to characterize the PU domains. Co was used as a source for both experiments. The angle sweeps for the SAXS and WAXS were from 0.1° to 5°, and 4° to 90°, respectively. The patterns were recorded over a zero-background holder. The interdomain space (d) was calculated using Bragg’s law, and the following equation:

d = 2π/q_max_ (4)

where q_max_ is the peak position of the SAXS patterns.

A TGA was conducted in two steps using a TGA/DSC 1 (Mettler Toledo, Columbus, OH, USA). First, the samples were heated for 1 h at 105 °C to remove water and solvent traces. Then, the samples were dynamically heated at 10 °C/min from 105 °C to 600 °C under a nitrogen atmosphere.

## 3. Results and Discussion

**Figure 1:**
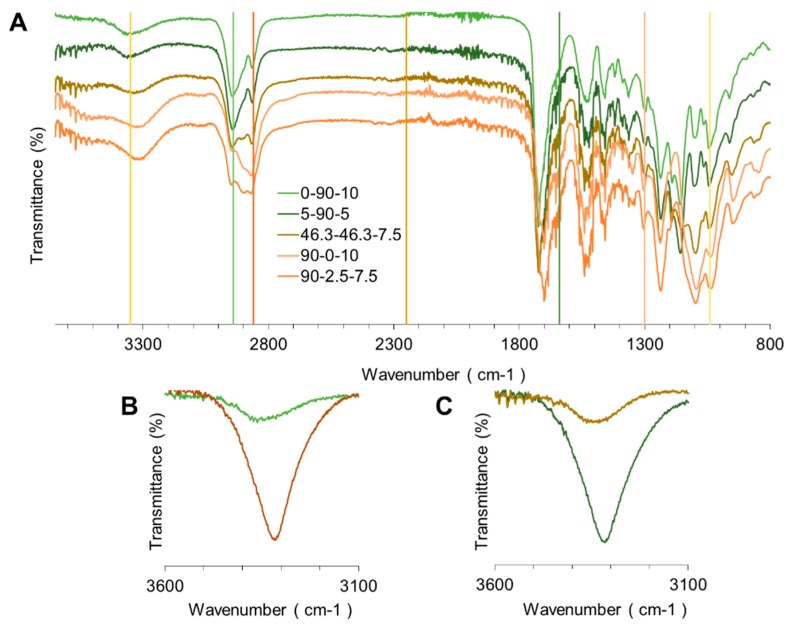
Fourier transform infrared spectra: (**A**) representative spectra of PUs, (**B**) spectra of the 0-90-10 sample recorded at two different positions, and (**C**) spectra of 2.5-90-7.5 sample recorded at two different positions.

The successful PU synthesis was evaluated by FTIR. Figure 1A shows some of the PU spectra. Characteristic vibrations of urethane bonds were observed around 1730 cm^−1^ from the C = O stretching, at 1300 cm^−1^ and 1040 cm^−1^ from the -C-O-C- asymmetric and symmetric stretching, and at 1550 cm^−1^ and 3350 cm^−1^ from the -N-H-. Additionally, peaks around 2940 cm^−1^ and 2860 cm^−1^ for the asymmetric and symmetric vibrations of -CH_2_, respectively, were observed. Finally, the broadband of primary -OH from PEG, PCL, and PE, around 3600 cm^−1^ and the NCO peak at 2250 cm^−1^ were identified. The PUs were successfully synthetized, and the NCO/OH ratio of 1 allowed for the complete reaction between polyols, the crosslinker, and the isocyanate.

During the evaluation of the spectra results, we identified variations between them. Between the wavelength of 3300 cm^−1^ and 3600 cm^−1^, as seen in Figure 1A, a shoulder-like structure is observed, which is easier to identify as PEG is increased in the figure. The -N-H in the urethane bonds can be either free or associated [11]; the former can contain two bands, one related to the associated groups and one related to the non-associated ones. Brzeska et al. [3] described similar behavior in branched PUs obtained from polycaprolactonetriol and synthetic polyhydroxybutyrate (PHB). They ascribed the shoulder-like structure to the non-associated -N-H groups when PHB content was increased. Similarly, Tan et al. [12] assigned a free -N-H group to a higher wavelength than that of the bonded groups. In this study, the shoulder-like structure decreased, which represents an increase in PEG content and a reduction in PCL. Moreover, an increase in PEG could reduce the physical crosslinking between the urethane bonds. Additionally, the spectra evaluation revels changes in the -N-H vibrations. 0-90-10 and 2.5-90-7.5 displayed sharper and broader peaks in some spectra. As previously mentioned, this peak is related to the associated urethane bonds, suggesting an increase in the interactions between the HS. The PU segmented structures have been reported on as thermodynamically incompatible, producing insolubilities, and phase separation [13]. Shaper peaks could be related to the presence of the HS domain at the point where the spectrum was measured. This phenomenon can be observed in Figure 1B,C.

The calculated DPS (See Table 2) did not show statistical difference between PU composition. Thus, DPS could not be used as a tool for measuring the phase separation. However, values of standard deviation for 0-90-10 and 2.5-90-7.5 are larger than other Pus, supporting the aforesaid findings in Figure 1B,C. A large deviation could be caused by sharpened of -NH vibration due to increase of hydrogen bounding interaction.

Around the HI (See Table 2), previous reports explain that larger hydrogen bounding enhances phase mixing as a result of less soft chain mobility [14]. Lower values were reached by the 90-0-10 and 90-2.5-7.5, which each other material have significant differences indicating poor phase mixing for those. Likewise, the other six materials showed larger values of HI, suggesting better phase mixing. Again, the HI did not correlate with the HS contents implying there are other phenomena involved in the crosslinking like: the molecular weight and nature of polyols or functional groups accessibility [15].

**Figure 2:**
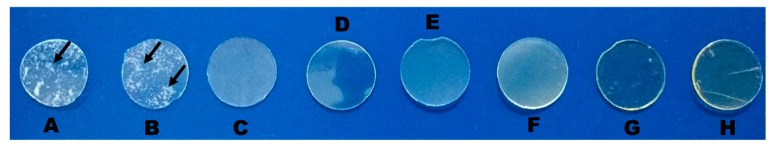
Example of the eight PEG/PCL/PE blends A) 0-90-10, B) 2.5-90-7.5, C) 5-90-5, D) 45-45-10, E) 46.3-46.3-7.5, F) 47.5-47.5-5, G) 90-0-10, and H) 90-2.5-7.5. The arrows in A and B point to the opaque regions.

Figure 2 shows the eight synthesized PU blends. Figure 2A,B corresponds to the 0-90-10 and 2.5-90-7.5 samples, respectively. A nonhomogeneous distribution with clear and opaque regions (highlighted by the arrows) was observed in the materials, with the other six PUs (Figure 2C–H) displaying the opposite behavior. The opaque regions in the PUs could be due to partial crystallisation because domain organization can lead to HS and SS crystallization [3]. This behavior could explain the sharpness of the -N-H vibrations in the same PUs.

**Figure 3:**
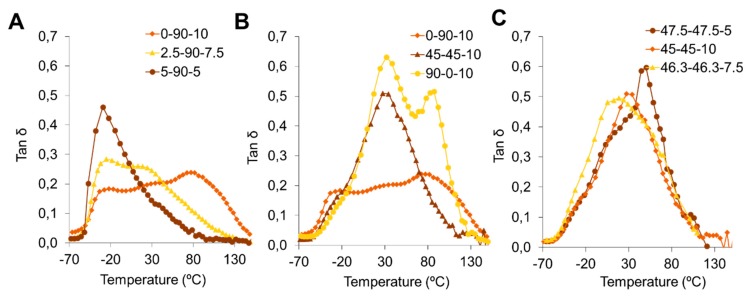
Loss factor of the synthesized PUs: (**A**) comparing changes in crosslinker, PE, content; (**B**) comparing changes in polyol composition; and (**C**) comparing blends with an equal ratio of polyols.

The 0-90-10 and 2.5-90-7.5 samples are composed in a large part by PCL, a polyester, and crosslinkers. However, they do not have the highest HS content (See Table 2). The high polarity of the urethane bonds promotes phase separation. According to Imre et al. [13], segment miscibility increases with an increase in SS polarity. PCL is a well-known hydrophobic polymer, and it can drive phase separation, which explains the FTIR behavior described previously. The increase in PEG content can also enhance segment miscibility while leaving some free, as shown in Figure 3A.

The thermal analysis provided information about molecular interactions and phase separation. The loss factor, from the DMA, described the transitions that occurred in the materials during heating, where each maximum peak temperature corresponds to a transition temperature. Figure 3 shows the loss factors of several PU blends obtained by the DMA. For some cases, we observed multiple peaks, indicating that some PUs present more than one phase and are non-homogenously distributed. The PUs are composed of SS and HS; therefore, we propose three main interactions and possible domains in the PU matrix: soft-soft, hard-hard, and soft-hard.

Figure 3A compares PUs with the largest concentration of PCL and the three compositions of PE. Larger concentrations of PE, and larger HS contents according to Table 2, generate more transitions in the PUs and, therefore, more than one peak. This may be an indicator that there is more than one phase or domain in the PU. Moreover, the reduction in PE means an increase in PEG and a reduction in the number of peaks; the 0-90-10 sample has three peaks, the 2.5-90-7.5- sample has two peaks, and the 5-90-5 sample has one. This supports the theory that an increasing polarity of SS may improve phase mixing. Additionally, Klinedinst et al. [4] found that a longer HS and SS may increase SS-HS incompatibility and may lead to less interaction between HS and SS domains, increasing the level of microphase separation. As described in the materials section, PEG has a lower molecular weight than PCL. A reduction in the average molecular weight may lead to additional phase mixing and reduces the crystallization of SS at low temperatures. This observation is in agreement with the observations from FTIR, where we identified associations between HS. For 0-90-10, the lowest peak temperature is assigned to the transition of SS while the largest is assigned to HS.

Figure 3B compares polyol blends with a fixed concentration of PE. The sample with an equal ratio of PCL and PEG (PCL/PEG = 1) displayed one peak while the other two samples, corresponding to the maximum concentration of PCL and PEG, displayed more than one peak, indicating that the extreme compositions generate domains in the PU structures.

The below behavior, of 90-0-10 related with the HI findings where those polymers had lower values inferring poor phase mixing. However, for 0-90-10 and 2.5-90-7.5, data did not match. Values of HI were largest and similar to other PUs but described a different behavior on DMA analysis, meaning other phenomena are involved in the phase separation and not only crosslinking drive a better phase mixing. In fact, the relation between crosslinking and phase separation is still unclear [10].

For the three blends with PEG/PCL = 1 and different concentrations of PE, as presented in Figure 3C, we observed that equal amounts of PEG and PCL produce one broad peak of loss factor. The broadening loss factor peak may indicate a reduction in the amount of chain movement. For these cases, phase mixing can be improved by generating a structure were SS movements are restricted by the HS and their interactions. This is analogous to the work reported by Lei et al. [16], where the incorporation of nanocrystalline cellulose in a PU matrix restricted chain movements because the cellulose acts as a reinforcement agent and crosslinker. The HS in the PUs can reduce SS mobility since they are composed of IPDI and PE, where PE induces crosslinking during synthesis at a functionality level of four. In this way, the largest concentrations of HS have the largest values of storage modulus.

Lei et al. [17] mention that the width of a loss factor peak represents an increase in the interface volume that can improve the PU internal friction and damping capacity. The areas under the curve of the loss factor for all PUs are reported in Table 3. The area under the curve was calculated using the trapezoid method and was used as a measurement of the wide loss factor peak [18]. Comparing these results with those in Figure 3, we can infer that the largest values of area under the curve represent the widest curves. Increasing the PEG content improves the damping capacity of PUs, and a PEG/PCL ratio equal to one produce PUs with the highest damping capacity and best phase mixing.

Table 3 also shows the SS Tg. Tg was calculated as the temperature of the maximum value of the loss modulus, as described by Huda et al. [19]. The SS Tg can be determined from either the loss factor or loss modulus peak. Tg moves slightly to the left when the PE content is reduced. Based on Table 2, reducing the PE content also decreases the HS content. Therefore, PUs with lower HS contents allow chain movement and Tg displacement to lower values.

**Figure 4:**
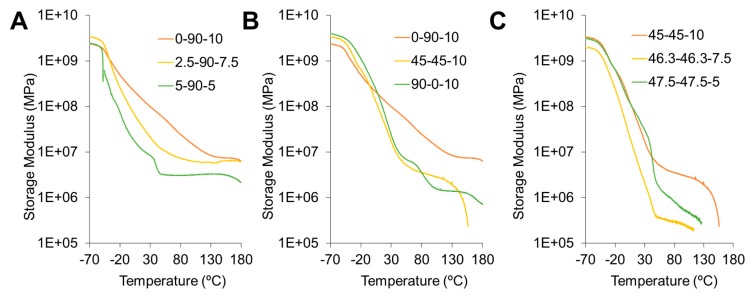
Storage modulus comparing (**A**) changes in crosslinker, PE, and content; (**B**) changes in polyol composition, and (**C**) blends with an equal ratio of polyols.

Figure 4 shows the storage modulus for all Pus, where the three stages were identified. It illustrates a glassy plateau region followed by a decrease in storage modulus, a leathery region. Finally, there is a rubbery plateau. The PUs follow typical elastomeric behavior. For these cases, a second drop of the storage modulus relating to viscous flow was identified for samples with the largest amounts of PEG. In Figure 4A, it can be observed that the modulus decreases when the PE amount decreases, which can be attributed to a lower crosslinking. Furthermore, increasing PEG content decreases the modulus, as shown in Figure 4. Finally, similar behavioral PE concentration effects are observed in Figure 4C.

**Figure 5:**
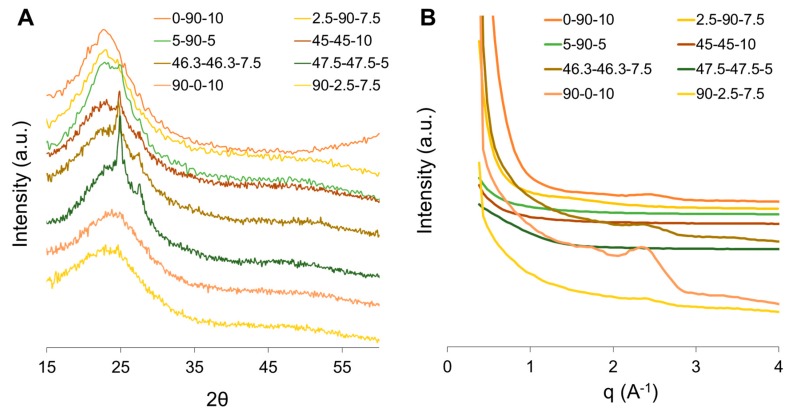
X-ray scattering patterns: (**A**) WAXS and (**B**) SAXS for synthetized PUs.

X-ray scattering was used to study PU morphologies. Figure 5A shows the WAXS profile of the PUs. Most of the samples displayed a broad scattering halo with maxima centering 23° < 2θ < 24°, suggesting the amorphous form of PUs. However, PUs with PEG/PCL = 1 showed a sharp peak at the top of the broad halo. According to Ti et al. [20], this reveals the existence of ordered microstructures that could be related to the tightly-packed HS. Additionally, there is an extra peak centering around 2θ = 27° that can be attributed to a SS crystalline region [17]. In contrast, Figure 5B showed the SAXS profiles. PUs with the largest amounts of PCL and PEG (0-90-10, 2.5-90-7.5, 90-0-10, and 90-2.5-7.5) exhibit an interference peak in the scattering plot. Applying Bragg’s law, the interdomain space was calculated only for the PUs mentioned above, as shown in Table 3. The spacing values are small compared with those reported in the literature. Kim, H-N., et al. [1] reported values of 12 nm and 20 nm for PUs synthetized from poly (tetramethylene ether glycol) as the polyol, BD, ISB and IMN as the chain extenders while Ti et al. [20] reported spaces between 4.57 nm and 7.95 nm in polyureas obtained from diaminopolypropylene glycol as the polyols, IPDI, and hexamethylene diisocyanate, and bis(4-aminophenyl)disulfide and 4,4′-diaminodibenzyl as the chain extenders. Despite these findings, the distances observed in this study are similar to those described by Cao and Liu [21].

**Figure 6:**
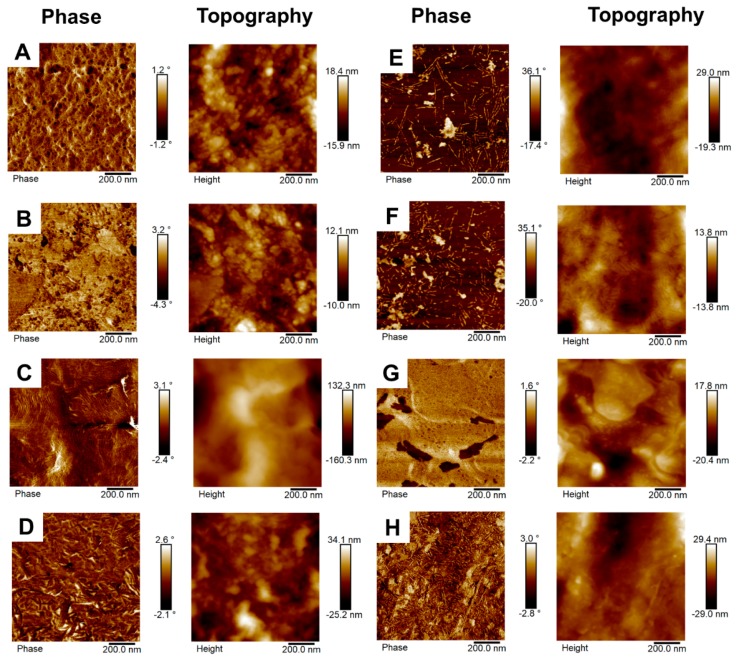
AFM phase and topography images of synthetized PUs, using the tapping mode (**A**) 0-90-10, (**B**) 2.5-90-7.5, (**C**) 5-90-5, (**D**) 45-45-10, (**E**) 46.3-46.3-7.5, (**F**) 47.5-47.5-5, (**G**) 90-0-10, and (**H**) 90-2.5-7.5.

To identify phases in PUs, AFM was carried out. Figure 6 showed the phase and topography images obtained for the eight PUs. Topography images revealed nonuniform surfaces for all samples. Phase images where used to study the rough surface—phase images produce greater detail, which are not easily detected by topographical imaging and, also, phase contrast arises from compositional variations of the surface [22], which allows identifying hard and soft segments. We can observe brighter and darker regions. Lu et al. explained HS had higher modulus showing brighter areas, while SS had lower modulus exhibiting darker regions [23].

It can be easily inferred that polyol/crosslinker blend composition affects PUs microphase separation and structures. In Figure 6A, 0-90-10 showed a sphere-like structure with large bright regions. Similarly, 2.5-90-7.5 (Figure 6B) described large bright regions with some dark dots. HS arranged in broad phases, which agrees with the FTIR spectra where those PUs had domains with more associated HS. Also, DMA proves the presence of more than one phase due to insolubility between the PUs segments.

The addition of PEG to the PUs, which increase the hydrophilic nature of SS, generated changes in phase structure. Hence, 5-90-5 (Figure 6C) described a thread-like and bright structure dispersed in the dark regions ergo HS dispersed in SS. Likewise, 45-45-10, 46.3-46.3-7.5, and 47.5-47.5-5 (Figure 6D–F respectively) showed thread-like structure but shorter than 5-90-5. Additionally, bright granules can be observed, indicating that more than one structure composed by HS can exist. Also, those images described better phase mixing, which agrees with the monomodal diagram of Tan δ found at DMA. 45-45-10, 46.3-46.3-7.5, and 47.5-47.5-5 reveals HS dispersed in SS and the largest dark areas and from DMA records, 45-45-10, 46.3-6.3-7.5 and 47.5-47.5-5 described some of the greatest values of area under the curve (See Table 3), which is related with largest damping capacity. That relationship is explained by Shön, P. et al. who mentioned that SS have larger energy dissipation than HS, or more crystalline structures [24].

Finally, Figure 6G,H corresponds to 90-0-10 and 90-2.5-7.5, respectively. They showed a broad and extensive HS domains. Like 0-9010 and 2.5-90-7.5, it demonstrated the insolubility in these PUs and supports the findings in the DMA.

**Figure 7:**
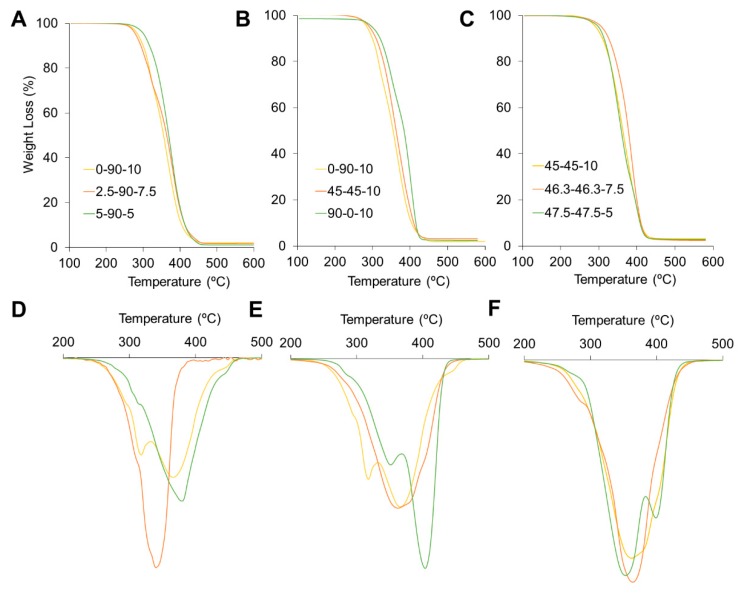
Thermogravimetric analysis of synthetized PUs: (**A**–**C**) corresponds to weight loss against temperature curves, while (**D**–**F**) corresponds to the first derivate of weight loss curves. (**A**,**D**) comparing changes in crosslinker, PE, content, (**B**,**E**) comparing changes in polyol composition, and (**C**,**F**) comparing blends with an equal ratio of polyols. DTGs corresponds to the top weight loss curves.

Figure 7A–C presents the weight loss curves obtained by TGA. PUs were thermally stable under ca. 230 °C and complete degradation were reached out over 450 °C. The first derivate of the thermogravimetric (DTG) curves are presented in Figure 7D–F. PUs with larger concentrations of PCL are shown in the figure, and the three main degradation steps are also presented. The first step is around 300 °C, the second is around 400 °C, and the final one is over 400 °C. In the past, these degradation steps have been assigned to the segmented PU structures. The first step is assigned to the thermal degradation of urethane bonds, while the second corresponds to SS degradation, and the third is related to the final decomposition of residue [25]. For the 0-90-10 sample, the first two stages were identified as two peaks, but the other two polymers showed a shoulder-like structure instead. This could be handled by the phase separation and the segment concentration. Partially separated peaks on DTG curve implies a significant degree of phase mixing as a result of enhanced segment interaction [26]. Thus, as the results of DMA, the incorporation of PEG helps with phase mixing. Also, in Figure 7B, 0-90-10 and 90-0-10 described separated peaks, which agree with the finding of DMA where more than one transition was observed. Also, 45-45-10 described one peak of thermal degradation inferring enhanced phase mixing. Besides, Figure 7C showed PUs with PEG/PCL = 1. PUs described one peak except for 47.5-47.5-5, which also described a shoulder-like structure in Figure 3C. Hence, thermal degradation confirms the behavior described by DMA and AFM. Incorporation of PEG improves phase mixing in polyurethanes.

As shown in Table 2, a reduction in PE in PUs decreases their HS content. Together with segment domains, this could produce overlapping degradation steps. We analyzed the temperature and maximum degradation rate. Figure 7A, *T*_max_ decreases as PE decreases. A reduction in PE can also reduce the chemical crosslinker and the thermal stability of the materials. The 47.5-47.5-5 sample exhibited a lower temperature than that of the 45-45-10 sample.

## 4. Conclusions

PUs were obtained using PE as the crosslinker. FTIR spectra confirm the synthesis of the urethane bonds. Addition of PEG enhanced phase mixing, however larger concentrations of crosslinker leads to HS segregation affecting properties and morphology of PUs. So, larger concentrations of PE generated larger phase separations and were observed in a multimodal loss factor pattern, while PUs with PEG/PCL = 1 showed a monomodal microphase distribution with packed HS. The incorporation of PEG in PUs improves phase mixing, as observed by the DMA. The SAXS analysis determined that some polymers with larger concentrations of PEs had branched structures and small interdomain spaces suggesting nanodomains. AFM phase images showed agglomeration of HS in PUs like 90-0-10 and 0-90-10. Also, PUs with PEG/PCL = 1described thread-like structure of HS and better phase mixing. Finally, the thermal stability of the studied PUs were affected by the concentration of polyols and the crosslinker.

## Figures and Tables

**Table 1:** Polyol/crosslinker blend compositions.

PU Name	PEG (% w/w)	PCL (% w/w)	PE (% w/w)
0-90-10	0	90	10
2.5-90-7.5	2.5	90	7.5
5-90-5	5	90	5
45-45-10-	45	45	10
46.3-46.3-7.5	46.3	46.3	7.5
47.5-47.5-5	47.5	47.5	5
90-0-10	90	0	10
90-2.5-7.5	90	2.5	7.5

**Table 2:** Theoretical HS content, DPS, HI of the eight synthesized PU blends.

PU Name	% HS	DPS *	HI *
0-90-10	36.90	45.62 ± 5.18	117.20 ± 18.3 ^a^
2.5-90-7.5	31.51	45.18 ± 5.09	112.85 ± 2.88 ^a^
5-90-5	25.47	49.17 ± 0.34	103.55 ± 6.26 ^a^
45-45-10	39.22	49.46 ± 0.13	110.36 ± 6.34 ^a^
46.3-46.3-7.5	34.09	49.66 ± 0.08	116.66 ± 1.29 ^a^
47.5-47.5-5	28.36	49.22 ± 0.13	126.87 ± 9.29 ^a^
90-0-10	41.37	48.80 ± 0.55	70.63 ± 5.42 ^c^
90-2.5-7.5-	36.48	48.30 ± 0.08	67.36 ± 0.07 ^d^

* DPS and HI are presented as the mean value ± standard deviation. Values with different letter means significant differences (*p* < 0.05).

**Table 3:** Glass transition (Tg) and area under the curve of the loss factor for the synthesized PUs, Interdomain space (d) from SAXS patterns, and temperature with maximum degradation rate (Tmax) from the TGA.

PU Name	Tg (°C)	Area under the Curve (1/°C)	d (A^−1^)	Tmax (°C)
0-90-10	−36.7	20.7	2.64	360
2.5-90-7.5	−42.5	20.3	4.15	341
5-90-5	−43.8	17.1	-	380
45-45-10	−37.7	29.4	-	366
46.3-46.3-7.5	−37.9	47.8	2.80	365
47.5-47.5-5	−39.5	39.6	-	354
90-0-10	−32.9	36.5	2.69	406
90-2.5-7.5	−33.8	52.2	2.54	402
